# Sensor Validation and Diagnostic Potential of Smartwatches in Movement Disorders

**DOI:** 10.3390/s21093139

**Published:** 2021-04-30

**Authors:** Julian Varghese, Catharina Marie van Alen, Michael Fujarski, Georg Stefan Schlake, Julitta Sucker, Tobias Warnecke, Christine Thomas

**Affiliations:** 1 Institute of Medical Informatics, University of Münster, 48149 Münster, Germany; michael.fujarski@uni-muenster.de (M.F.); georg.schlake@uni-muenster.de (G.S.S.); j_suck01@uni-muenster.de (J.S.); 2 Institute of Geophysics, University of Münster, 48149 Münster, Germany; c_vana01@uni-muenster.de (C.M.v.A.); cthom_01@uni-muenster.de (C.T.); 3 Department of Neurology, University Hospital Münster, 48149 Münster, Germany; tobias.warnecke@ukmuenster.de

**Keywords:** smartwatches, artificial intelligence, movement disorders, Parkinson’s disease

## Abstract

Smartwatches provide technology-based assessments in Parkinson’s disease (PD). It is necessary to evaluate their reliability and accuracy in order to include those devices in an assessment. We present unique results for sensor validation and disease classification via machine learning (ML). A comparison setup was designed with two different series of Apple smartwatches, one Nanometrics seismometer and a high-precision shaker to measure tremor-like amplitudes and frequencies. Clinical smartwatch measurements were acquired from a prospective study including 450 participants with PD, differential diagnoses (DD) and healthy participants. All participants wore two smartwatches throughout a 15-min examination. Symptoms and medical history were captured on the paired smartphone. The amplitude error of both smartwatches reaches up to 0.005 g, and for the measured frequencies, up to 0.01 Hz. A broad range of different ML classifiers were cross-validated. The most advanced task of distinguishing PD vs. DD was evaluated with 74.1% balanced accuracy, 86.5% precision and 90.5% recall by Multilayer Perceptrons. Deep-learning architectures significantly underperformed in all classification tasks. Smartwatches are capable of capturing subtle tremor signs with low noise. Amplitude and frequency differences between smartwatches and the seismometer were under the level of clinical significance. This study provided the largest PD sample size of two-hand smartwatch measurements and our preliminary ML-evaluation shows that such a system provides powerful means for diagnosis classification and new digital biomarkers, but it remains challenging for distinguishing similar disorders.

## 1. Introduction

Smart devices are broadly used in everyday life with many use cases for classification tasks, e.g., human activity recognition via wearable sensors, smart phones or cameras [1,2,3]. In addition, there are emerging research applications for different diseases—in particular, movement disorders [4]. Our work focuses on smartwatch-based analyses in diagnostic research of Parkinson’s disease (PD). It is the second-most neurodegenerative disorder—following Alzheimer dementia—and worldwide burden has more than doubled over the last two decades [5]. Early and accurate diagnoses improve quality of life and reduce work losses, which is why missed diagnoses mean missed opportunities [6]. Currently, PD diagnosis is primarily based on clinical assessment, which is challenging and associated with overall misclassification rates of around 20 to 30%; Rizzo et al., 2016 conducted a meta-analysis and reported pooled diagnostic accuracy of 73.8% for general practitioners or general neurologists with a 95% credible interval (CRI) of 67.8 to 79.6%.

Clinical assessment may not identify subtle changes in movement pathologies as, e.g., weak tremor, its frequency or slowness of movement [7]. Regarding diagnostic accuracy and treatment monitoring, there is a strong need for new technological objective biomarkers that are capable of capturing these subtleties with high precision and are machine-readable [4]. In the era of the digital transformation of healthcare, consumer wearables with multi-sensor technology provide a source of objective movement monitoring, allowing for greater precision in recording subtle changes, unlike current clinical rating scales in hospital routine [8]. Though there is an increasing number of such wearables and mobile apps or even mature medical devices, such as the Parkinson’s KinetiGraph^TM^ system by Global Kinetics, Melbourne, Australia [9], there is a low number of large-scale deployments [10].

Regarding PD, some systems have shown promising diagnostic potential when analyzing voice, hand movements, gait, facial expressions, eye movements and balance [11,12,13,14,15,16,17]. Most of these promising examples have used machine learning approaches for disease classification. However, the reported accuracies need to be taken with high caution because the implemented models were trained and tested on low sample sizes regarding PD (n < 100), which carries a high risk of overfitting. Moreover, we could not find any approach that includes similar movement disorders as an important control group for differential diagnoses. A simple classification model that only differentiates between PD and healthy controls is of only limited clinical use as it was only trained and tested between those classes and thus might have only learned to identify general movement anomalies, which differ from the healthy population but do not represent Parkinson-specific features. This is a common problem in binary classification, where the two classes are note exhaustive, e.g., healthy vs. not-healthy is exhaustive. PD vs. healthy is not exhaustive as there are many diseases that are not PD and not healthy. For example, there are diseases similar to PD that show almost the same symptoms. Hence, such models could misclassify other movement disorders such as multiple sclerosis or essential tremor. Moreover, in clinical reality, the health practitioner or the neurologist cannot initially assume whether the patient is either healthy or has PD. Therefore, classification models for potential diagnosis should consider differential diagnoses.

Our research focuses on acceleration-based hand movement analyses using a smart device system (SDS) that utilizes two smartwatches and a smartphone to distinguish PD from other movement disorders and healthy participants [18]. The study has recruited and measured > 400 participants and has generated one of the largest databases for PD, differential diagnoses and healthy subjects with acceleration data from a neurological examination including the left and right side of the body and structured clinical data on non-motor symptoms (e.g., sleep disturbances, loss of smell, depression). The system includes simple consumer devices by Apple, utilizing smartwatches to capture acceleration and a paired smartphone for clinical data. To our knowledge, official information on the smartwatch raw measurement accuracy is not publicly available. Therefore, the devices were evaluated by a systematic comparison with a gold standard utilizing a broadband seismometer.

Apart from this sensor validation, the SDS is integrated into a neurological examination. It consists of 10 steps to monitor and provokes specific movement characteristics such as tremor or slowness of movement. While the study is still running until the end of 2021 and includes further smart device data such as tablet-based drawing and voice analyses, this manuscript aims to focus on the following research aims:

- Sensor validation to measure the precision of smartwatches regarding acceleration amplitudes and tremor frequencies. As a gold standard, we conducted a comparison experiment utilizing a seismometer and a high-precision shaker. As a result, we assessed the level of precision regarding the smartwatches. This is particularly useful in the case of subtle tremors, which have acceleration amplitudes of < 0.05 g and are hard to capture by human vision.
- Timeseries features were extracted based on expert-based feature engineering and literature data. A broad range of machine learning models was trained and cross-validated to assess classification performances. To complement the expert-based feature engineering by a pure automatic feature extraction method, a deep-learning neural network with the raw time series data as input was trained and cross-validated as well.

The unique contribution of our work is a sensor validation experiment comparing consumer smartwatches to a gold standard seismometer and to evaluate machine learning models to assess the diagnostic potential based on one of the largest prospective examination studies that integrated smartwatches.

## 2. Materials and Methods

### 2.1. Overview of Data Processing Steps

**Figure 1:**
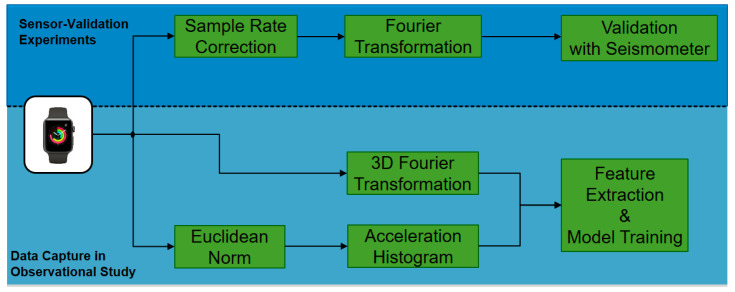
Processing steps include smartwatch validation with seismometers and patient data generation via an observational study for diagnostic machine learning.

The smartwatch validation experiments were carried out during the human subject trial. The trial generated the acceleration and questionnaire-based data in clinical examinations. Figure 1 provides an overview. The following section *Study Data Generation* introduces into the human subject trial, which generates data for the machine learning task of disease classification. The section *Smartwatch Sensor Validation* details the validation experiment with seismometer. The section *Machine Learning Pipeline and Features* describes data processing steps for the disease classification task. In particular, Table 3 and Figure 3 provide a deeper insight into the data features and technical machine learning steps.

### 2.2. Study Data Generation

The prospective study started in 2018 and was extended till the end of 2021. It received approval by the ethical board of the University of Münster and the physician’s chamber of Westphalia-Lippe (Reference number: 2018-328-f-S). It is being conducted at the outpatient clinic of movement disorders at the University Hospital Münster in Germany. The details of the study design and the protocol have been published previously [18]. Study registration ID on ClinicalTrials.gov: NCT03638479.

Table 1 lists participants population characteristics. Further information on demographics, differential diagnoses is provided for each sample in the Supplementary Patient-Population. All diagnoses were confirmed by neurologists and finally reviewed by one senior movement disorder expert.

Each participant wore two smartwatches, one on each wrist, while seated in an armchair and following a pre-defined neurological examination, which was instructed by a study nurse. This examination was designed by movement disorder experts with the primary aim to establish a simple-to-follow examination in order to capture the most relevant acceleration characteristics. The data consists of the acceleration data recorded by the smartwatches and further clinical data containing non-motor symptoms recorded on the paired smartphone. The non-motor symptoms are based on the Parkinson’s Non-motor Symptoms Questionnaire [19]. Each examination took 15 min per participant on average. Each assessment step is summarized is Table 2. The data-capturing app, which connects all devices, is installed on the smartphone. It is an in-house developed iOS-based research app [20] and will be provided as open source after the end of the study.

### 2.3. Smartwatch Sensor Validation

A seismometer is a device that captures weak ground motion caused by seismic sources, e.g., earthquakes, explosions or ambient noise [22]. These instruments generally have a large bandwidth and dynamic range [23]. The Trillium Compact by Nanometrics, Milpitas, CA, USA is a triaxial seismometer, measuring ground velocity and classified as a broadband instrument with −3 dB points at 120 s and 108 Hz. The self noise level is below −140 dB and the clip level at 26 mm/s up to 10 Hz and 0.17 g above 10 Hz [24]. We combined the Trillium Compact with a Taurus 24-bit digital recorder [25], which digitizes the motion that the seismometer measures. This combination allows for accurate measurements of ground motion [26] and is therefore considered as a gold-standard instrument for raw measurements of acceleration.

**Figure 2:**
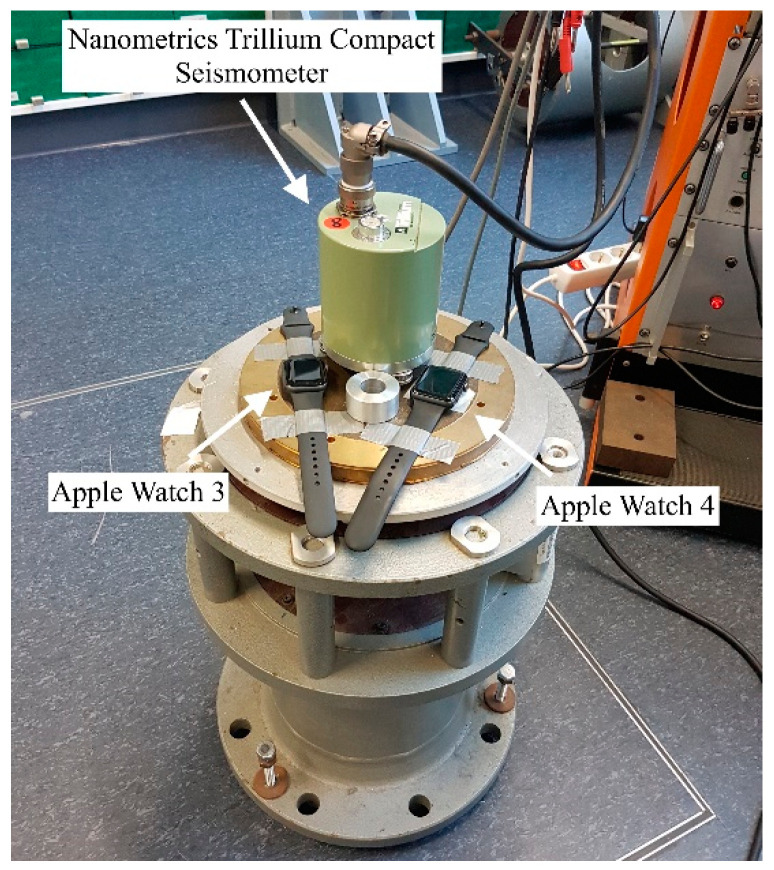
Experimental setup of the sensor validation experiment. Apple Watches Series 3 and 4 and a Nanometrics Trillium Compact seismometer were placed on a vertical vibration table. The table simultaneously accelerated the devices by oscillatory motions with tremor-typical frequencies and amplitudes. Both watches were connected to Apple iPhones (not in this figure) via Bluetooth, where the measurement data were stored. The seismometer data were collected on a digitizer (not in this figure) that the device was connected to.

We conducted a shaker table experiment, where two Apple watches, Series 3 and 4, and the Trillium Compact seismometer were simultaneously accelerated by oscillatory motions with tremor-typical frequencies and amplitudes. As tremor is an oscillatory movement, the use of a shaker table provides a means of testing accuracy of the method. The setup of the validation experiment is shown in Figure 2, where the seismometer and smartwatches were placed on a shaking table.

The watches were further attached with tape to prevent unwanted movement due to the slightly curved backside of the watches. The shaker table was placed on a decoupled platform to reduce ambient noise and oscillates vertically with a range of frequencies and amplitudes. Due to the experimental setup and since the vibration table moves in the vertical direction, only the *z*-axis of the watches and the seismometer was examined here. However, a significant difference in measurement accuracy between all three sensor components of the seismometer is not to be expected since the device records on three orthogonal axes U.V.W, which are then rotated into vertical and two horizontal components north and east [24].

The smartwatches are officially specified to have a sampling rate of 100 Hz and we set the sampling rate of the seismometer to 100 Hz as well. A total of 43 measurements were performed on two different days. The duration of each measurement was set to 20 s for the watches, similar to the assessment steps performed with patients.

For each test, the table oscillated with a set amplitude and frequency that was kept constant during the measurement period. One test was carried out without vibration, to measure the difference in self noise of the watches and the seismometer. For the remaining tests, we changed the frequency of the oscillation between 3 Hz and 15 Hz, in 1 Hz steps, as this range covers tremor-typical frequencies [27]. The oscillation amplitude was varied between 0.002 g and 0.1 g, which is considered as high-resolution for tremor amplitudes as values <0.01 g are barely visible by human vision but still clinically relevant to measure subtle tremor in early disease. The step sizes were between 0.0001 g and 0.02 g.

The data had to be processed after the experiments: First, the data of the seismometer were deconvolved with the instrument response. During the deconvolution, the counts per volts scaling factor of the raw data and the frequency-dependent sensor response were removed [28]. Since the seismometer records velocity while the watch records acceleration, the seismometer data were differentiated, converted from mm/s^2 to SI units and divided by 9.81 m/s^2, such that the output is in multiples of g, the Earth’s acceleration.

To determine the oscillation frequency for each 20 s measurement for both the seismometer and watches, the data were analyzed in the spectral domain, by applying the fast Fourier transform (FFT). The dominant frequency of each dataset was identified and compared. Prior to the FFT, the end of the data were zero-padded to reach a frequency bin spacing of 0.01 Hz because the frequency scale of the shaker table only allowed changes in in 0.01 Hz steps.

The oscillation amplitude was calculated in the time domain on the pre-processed datasets. For 20 consecutive periods, the maxima and minima of the signal were identified and used to calculate the peak-to-peak amplitudes. The resulting 20 peak-to-peak amplitudes were averaged and divided by 2. Subsequently, the results of the watches were compared to those of the seismometer in order to assesses the accuracy of the watches.

### 2.4. Machine Learning Pipeline and Features

Three relevant classification tasks were trained and cross-validated:

1. PD vs. healthy
2. Movement disorders (PD + DD) vs. healthy
3. PD vs. DD

It is assumed, that the first two tasks are of lower classification difficulty as the system only needs to be trained for non-healthy characteristics. Such a system could still be helpful in home-based settings or at general practices, e.g., to indicate whether certain abnormal movement characteristics (e.g., hand tremor) are pathologic or still normal (e.g., physiological tremor). The third one requires more advanced and differential feature analyses in order to distinguish movement disorders with similar phenotypical characteristics from each other.

**Figure 3:**
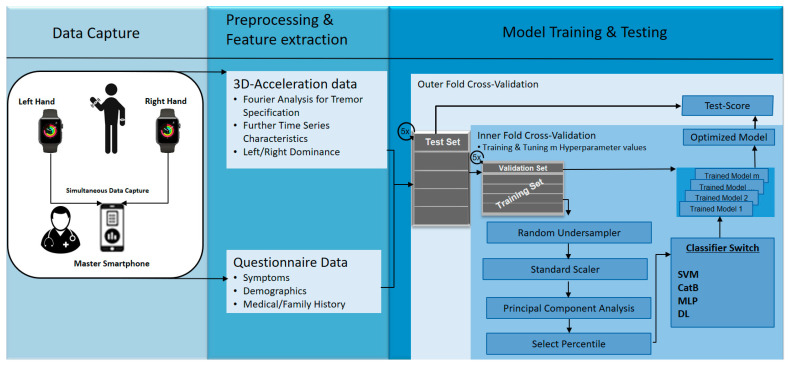
Overview of data analytics pipeline. SVM = support vector machine with radial basis function. CatB = CatBoost, MLP = multi-layer perceptron with two hidden layers, DL = deep-learning architecture.

The extracted features are listed in Table 3. We provide further details and pseudocode of feature extraction in the Machine-Learning Supplement. A previously developed Python-based data analytics pipeline is reutilized [20]. The entire analytics process is summarized and illustrated in Figure 3. The different machine-learning classifiers were support vector machines (SVM); a modern gradient-boosting decision-tree model called CatBoost [29]; a multilayer perceptron (MLP), which is a classical type of an artificial neural network; and a deep-learning architecture. These were trained and validated within the framework of nested cross-validation [30] using five outer and five nested inner data folds to ensure unbiased training and testing, as well as unbiased optimization of hyperparameters. While the inner folds are used to train each model and to optimize its hyperparameters in a grid-search (m different hyperparameter values results in m different model configurations), the outer folds evaluate the test performance of trained and hyperparameter-optimized models. Before each inner fold model training, we apply the random undersampler from Scikit Learn 0.24.1 [31] in order to remove the bias towards the majority class by randomly removing samples of that set. Moreover, the standard scaler from Scikit Learn subtracts the mean and scales to unit variance for every feature. The principal component analysis (PCA) reduces the dimensionality, the Scikit Learn-based ‘Select Percentile’ step randomly selects a subset of features, which are then used for training the classifier. We optimize the hyperparameters for the PCA, the Select Percentile and the specific classifiers. A detailed list of hyperparameter optimizations is provided in the Machine-Learning Supplement.

The multi-layer perceptron and the deep-learning architecture is implemented using Keras and Google’s Tensorflow 2.4.0, which provides full GPU support [32]. We considered various state-of-the-art architectures including convolutional neural networks in ResNets and long–short-term memories (LSTM) [33]. Detailed architectures are provided in the Machine-Learning Supplement.

To evaluate their performance for automatic time-series feature extraction from acceleration data, they only received the raw acceleration data and the questionnaire data (medical history + symptoms) as input, but not the engineered time-series features listed in Table 3.

Test performances for all three classification tasks are reported as mean values for precision, recall and F1-measure based on the outer-fold validations including standard deviations. Due to the imbalance of the three disease classes, balanced accuracies [34,35] are provided as well. As such, the baseline performance of all binary classification tasks is 50%, which corresponds to random guessing. To analyze the information gain of different features, we apply feature importance analyses via CatBoost for the second classification task as this involves all disease classes. Then, bootstrap sampling is applied to generate information gain boxplots for the different features.

## 3. Results

### 3.1. Smartwatch Sensor Validation

**Figure 4:**
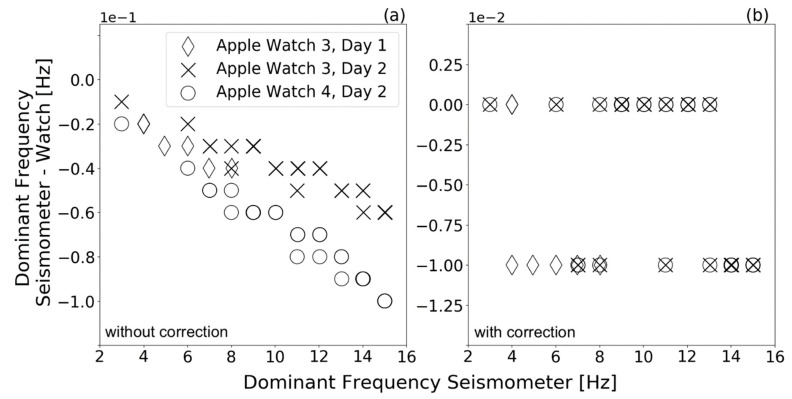
Differences between the dominant frequency measured by the Trillium Compact seismometer and Apple Smartwatches Series 3 and 4 in a shaker table experiment. The experiment was conducted on two different days with the Apple watch Series 3. The figure shows the difference in dominant frequency (**a**) using the pre-defined watches’ sample rate and (**b**) using the watches’ actual sample rate (calculated with watch-specific timestamps) for spectral calculations. Data points that have exactly the same value lie on top of each other in the plot. To show the effect of amplitude on these frequency differences, some measurements were repeated by keeping the shaking table frequency constant and varying the shaking table amplitude.

Figure 4a shows the differences between dominant frequencies of the seismometer (used as the gold standard device) and Apple Watches Series 3 and 4 data (consumer grade device). Overall, Apple watches Series 3 and 4 seemed to measure higher frequencies than the seismometer; however, deviations were in the low milli-Hertz range (up to 10 mHz). With increasing frequencies, there was an increase in frequency deviation for both watches and for all experiments.

As mentioned above, the watches’ sampling rates were set to 100 Hz. When calculating the watches’ sample rate using the watch-specific timestamps, however, we found that the sampling rates of the watches were up to 0.6 Hz smaller than the specified 100 Hz. We provide further details on time variations between two data points for both watches in the Machine-Learning Supplement (Supplementary Figure S7 and Table S8). The increasing deviations with increasing frequency therefore resulted from assuming an incorrect sample rate of 100 Hz for spectral calculations. Figure 4b shows the difference between dominant frequencies of the seismometer and smartwatches after correcting for the sample rate. For spectral calculation, the actual sample rate of the watches was used by utilizing the watch-specific timestamps. In the considered range, no clear increase in deviation with increasing frequency is recognizable anymore. Approximately 55% of the Series 3 and 59% of Series 4 dominant frequencies did not deviate from the seismometer up to the second decimal place. The remaining measurements deviated by up to 0.01 Hz for both Series 3 and 4. This still provides a high-precision tremor frequency capture, as clinical tremor documentation is performed in the range of 4 to 18 Hz and step sizes of full Hz units [27].

**Figure 5:**
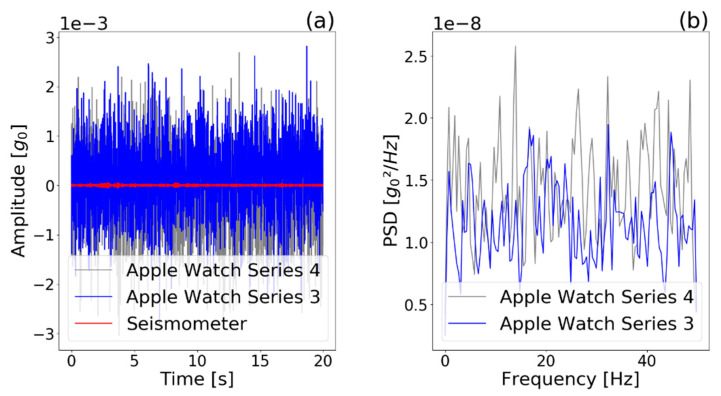
(**a**) Self noise of watches and seismometer and (**b**) power spectral density (PSD) of watches, captured during a 20-s period without vibration of the shaker table. The power spectral density shows that the noise of the smartwatches had a similar intensity at all frequencies covered. However, Apple Watch 4 had a slightly higher self noise.

We measured the self noise of the seismometer and the watches on the non-vibrating table. The results are depicted in Figure 5 and show that the watches had a higher noise compared with the seismometer, but the RMS self-noise level was still below 0.001 g for both watches. The 0 g-offset was found to be below 2 × 10^−4 g. The power spectral density shows that the noise of the smartwatches had a similar intensity at different frequencies.

**Figure 6:**
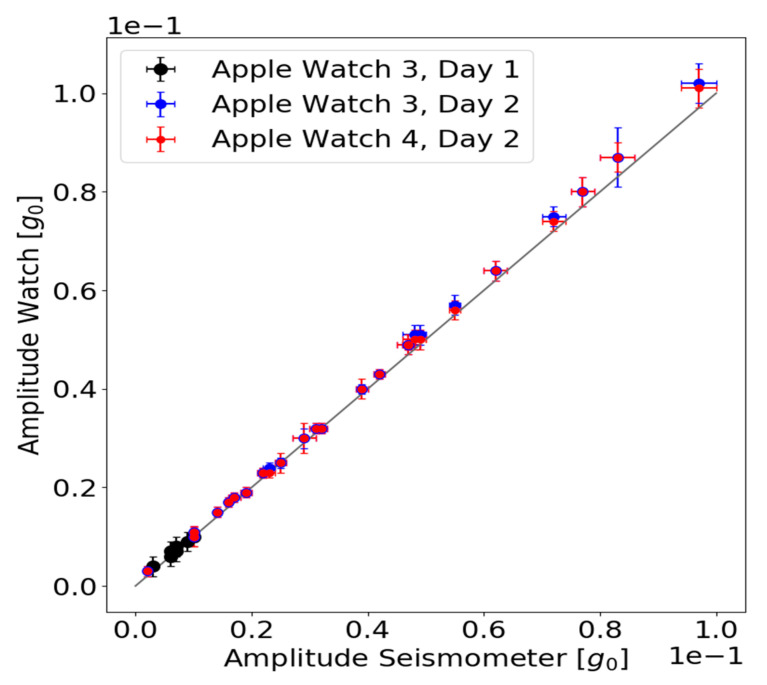
Measured oscillation amplitude of the seismometer and the watches are plotted against each other. The standard deviations of the amplitude mean values are plotted as error bars (horizontal error bar: seismometer values, vertical error bar: watch values). The grey line corresponds to a perfect agreement between the oscillation amplitude measured by the watches and the seismometer.

Figure 6 depicts the difference in measured oscillation amplitude for the seismometer and the smartwatches. For all the measurements, smartwatch Series 3 and 4 measured higher amplitudes than the seismometer. Up to 0.04 g oscillation amplitudes, the amplitude differences between the watches and the seismometer showed no trend and were below 0.002 g. Oscillation amplitudes >0.05 g led to larger deviations for both Series 3 and 4 and a trend is visible. We found the maximum deviation of 0.005 g. The amplitude measurements of the watches and seismometer agree within their corresponding standard deviations.

### 3.2. Classification Performances and Feature Importance

Table 4, Table 5 and Table 6 list model performances for all three classification tasks. Apart from the deep learning model, the other three classical machine learning models performed similar in respect to their standard deviations, with balanced accuracies above 80% and precision and recall above 90% in the two simpler classification tasks. Regarding the most difficult task, which required separation of Parkinson’s disease from similar movement disorders, all three models performed lower with balanced accuracies between 67% and 74%. The MLP performed best in two of three tasks (PD + DD vs. healthy, PD vs. DD) in terms of balanced accuracies.

**Figure 7:**
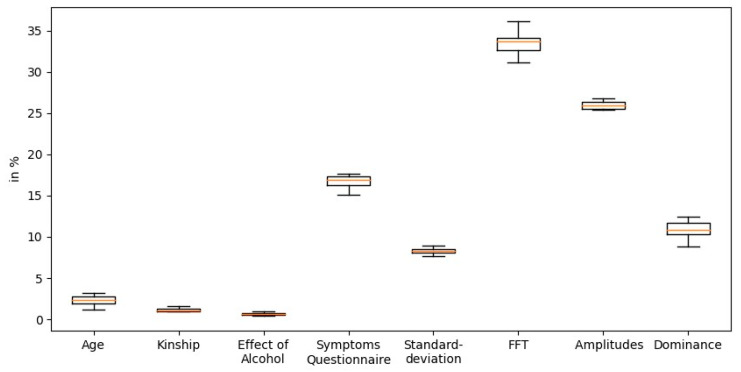
Importance of the features based on statistical information gain by CatBoost.

Figure 7 summarizes feature importance based on statistical information utilizing CatBoost. It shows that the highest overall gain is attributed to the sensor-based FFT features, while the symptoms questionnaires provide high gain among all questionnaire-based features.

Among the different combinations of DL architectures, the best-performing architecture included a simple dense neural network that could only reach balanced accuracies lower than 60%. It is noteworthy that the inclusion of LSTMs consistently weakened the classification performance and therefore did not participate in our final DL architecture. As the DL components underperformed in this complex task of diagnosis classification, we wanted to figure out how DL would perform in a simple activity recognition task, for which DL architectures are commonly applied. Thus, they were validated using the performed assessment steps as an activity recognition task (e.g., does time-series belong to assessment step 6, “drinking glass”?). Here, the best DL model performed with an accuracy of 78.6% with the ResNet. The same tasks reduced to the assessment steps ‘drink glass’ and ‘point finger’ even performed with an accuracy of 94.6% using DL architecture with simple dense neural networks. The detailed architectures for the DL models and their performances are provided in the Machine-Learning Supplement.

## 4. Discussion

The SDS is an app-based mobile system that connects consumer devices for the high-resolution monitoring of acceleration characteristics in different neurological disorders and questionnaire-based data capture of patient symptoms.

The seismological sensor validation showed high agreement between the smartwatches and the gold-standard setting. While clinical tremor documentation ranges between 4 and 18 Hz with step sizes of 0.1 to 1 Hz, the watches differed slightly from the gold standard at around 0.01 Hz. While the human tremor amplitude threshold can be estimated at <0.01 to 0.05 g [7], the smartwatch amplitude deviations were within the range of 0.001 and 0.005 g. This shows that the watches are capable of measuring movement subtleties or hand-tremor amplitudes and frequencies with much greater precision than clinical documentation or even human vision. We reproduced these findings with multiple measurements and two Apple-based smartwatch models of different build years.

When integrating two smartwatches and a paired smartphone to the SDS coupled with different AI-based classifiers, we could show high diagnostic accuracies, above 80%, partially with precision and recall above 90% for simple classification tasks. Related work shows even higher performances, consistently above 90% accuracy when using other data modalities, e.g., voice analyses [12]. However, while these findings doubtlessly show some diagnostic potential, they have to be interpreted with high caution as we believe these results are easily overestimated due to three key reasons: First, the overall sample size of almost all related studies were limited (n < 100). Second, model hyperparameters were not optimized in a separate nested set. Third, the same individuals were recorded multiple times, leading to identity confounding [36]. To address these frequent drawbacks and provide a higher degree of generalizability, we have generated—to the best of our knowledge—the largest database on this topic with more than 400 individually measured participants using nested cross-validation for all models and hyperparameters. In addition, we included the important control group of differential diagnoses. As expected, the most difficult task to separate PD from similar movement disorders was evaluated with much lower balanced accuracies of around 70%. This shows that further feature engineering and further integration of other promising modalities (acceleration, speech, voice or finger-tapping are needed. All these data modalities were studied in isolation with promising findings [4,12,37] and could be integrated within one system consisting of consumer devices. The results of our deep-learning architecture clearly show that automatic feature extraction is underperforming in this sample size dimension (n < 1000) and there is a strong need for engineering clinically relevant features in raw acceleration data.

A common limitation with related work, which is also not addressed by this study, is the missing evaluation of real predictive capabilities for early diagnosis as we can only include patients that have already been diagnosed or healthy participants, for which we do not know if they will develop a disease condition. Our study included a broad range of different disease progress states according to Hoehn and Yahr [38] or years from disease onset, but an observational epidemiological study with healthy-to-PD transformation data would be ideal to test disease prediction. Nevertheless, our work can provide potential features and methods, which need to be studied in future study designs to evaluate prediction performance. Moreover, our work contributes to new digital and objective biomarkers, which have the potential for disease stratification or disease monitoring of PD patients to provide personalized care and treatment optimization. As for all clinical decision support, further quality and risk management and medical device approval is necessary for integration into routine diagnostics [39]. To the best of our knowledge, our study generated the largest set of smartwatch-based measurements in a neurological examination with structured clinical data on symptoms and medical history. The anonymized raw acceleration and clinical data is going to be published after the end of the study (end of 2021). This unique dataset will enrich the current open repositories for the time series processing community and provide public access in order to enable further analyses beyond the research questions of this paper.

## Figures and Tables

**Table 1:** Participant population. DD: differential diagnoses including movement disorders other than PD as essential tremor, atypical Parkinsonism, secondary causes of Parkinsonism and dystonia, multiple sclerosis.

Disease Class	Sample Size	Average Age (SD)
PD	260	66.26 (9.61)
DD	101	60.82 (12.87)
Healthy	89	61.45 (10.63)

**Table 2:** Smartwatch-based examination steps.

Step	Duration (s)	Description
1a	20	**Rest tremor.** Participant is seated with his eyes closed in resting position, positioning standardized to Zhang et al. [21].
1b	20	**Rest tremor** while patient is calculating serial sevens.
2	10	**Lift and extend arms** according to Zhang et al. [21].
3	10	**Remain arms lifted.**
4	10	**Hold 1 kg** weight in each hand for 5 s. Start with the right hand. Then, have the participant’s arm rested again as in 1a.
5	10	**Finger pointing.** Participant should point with their index finger to examiner’s lifted hand. Start with participant’s right index, then left, then repeat.
6	10	**Drink from glass.** Have the participant grasp an empty glass with their right hand as if they would drink from it. Then repeat with the left hand.
7	10	**Cross and extend both arms.**
8	10	**Bring both index fingers to each other.**
9	10	**Let participant tap their nose with both index fingers.** Start with the right, then with left index. Then extend the arms.
10	20	**Entrainment.** The examiner stomps on the ground, setting the pace. The participant starts stomping with their right foot according to the pace while leaving their arms extended. Repeat this with the left foot.

**Table 3:** Machine Learning Features.

Feature	Description
Medical History Questionnaire	Age height, weight, family history of PD (kinship with PD), effect of alcohol on tremor. Further details provided in Varghese et al. [18]. Medication is captured but not used as a training-feature as it is too closely linked to the target classes.
Symptoms-Questionnaire	The number of items answered with ‘yes’ in the Parkinson’s disease Non-Motor Scale by the Movement Disorder Society [19].
Amplitude Distribution	Apply Euclidean norm on all three acceleration axes to generate 1-dimensional time-series vector. Create an Amplitude histogram and pick the 30th to 70th percentile in 5 percent steps. Applied for all assessment steps.
Tremor Side Dominance	Use the 90th percentile of the left and right arm acceleration and calculate the ratio. Applied for all assessment steps.
Standard Deviation of Acceleration	Calculate the standard deviation of the acceleration data. Applied for all assessment steps.
Fast Fourier Transformation	Calculate the three-dimensional FFT for the assessment step and use polynomials of degree 3 to approximate the FFT. The three coefficients are used as features. Applied for all assessment steps.

**Table 4:** Performances for classification task 1: separate PD from healthy. Values correspond to mean (SD). MLP = multi-layer perceptron, SVM—rbf = support vector machine—radial basis function, simple DNN = simple deep neural network.

Estimator	Accuracy	Balanced Accuracy	Precision	Recall	F1
MLP	0.864 (0.03)	0.815 (0.05)	0.907 (0.03)	0.913 (0.03)	0.909 (0.02)
SVM—rbf	0.870 (0.02)	0.827 (0.01)	0.913 (0.01)	0.913 (0.03)	0.913 (0.01)
CatBoost	0.887 (0.02)	0.819 (0.04)	0.901 (0.03)	0.956 (0.03)	0.927 (0.01)
Simple DNN	0.768 (0.06)	0.591 (0.07)	0.782 (0.03)	0.954 (0.06)	0.859 (0.04)

**Table 5:** Performances for classification task 2: separate movement disorders (Parkinson’s disease and differential diagnoses) from healthy. Values correspond to mean (SD). MLP = multi-layer perceptron, SVM—rbf = support vector machine—radial basis function, simple DNN = simple deep neural network.

Estimator	Accuracy	Balanced Accuracy	Precision	Recall	F1
MLP	0.856 (0.04)	0.772 (0.05)	0.907 (0.02)	0.914 (0.03)	0.910 (0.02)
SVM—rbf	0.838 (0.02)	0.750 (0.03)	0.901 (0.02)	0.897 (0.06)	0.897 (0.02)
CatBoost	0.882 (0.03)	0.757 (0.06)	0.895 (0.02)	0.968 (0.03)	0.929 (0.01)
Simple DNN	0.791 (0.03)	0.551 (0.06)	0.814 (0.01)	0.956 (0.03)	0.879 (0.02)

**Table 6:** Performances for advanced classification task 3: separate Parkinson’s disease from differential diagnoses. Values correspond to mean (SD). MLP = multi-layer perceptron, SVM—rbf = support vector machine—radial basis function, simple DNN = simple deep neural network.

Estimator	Accuracy	Balanced Accuracy	Precision	Recall	F1
MLP	0.823 (0.01)	0.741 (0.03)	0.865 (0.01)	0.905 (0.00)	0.885 (0.00)
SVM—rbf	0.800 (0.02)	0.682 (0.04)	0.831 (0.02)	0.921 (0.01)	0.873 (0.01)
CatBoost	0.817 (0.02)	0.678 (0.03)	0.826 (0.01)	0.956 (0.03)	0.887 (0.01)
Simple DNN	0.735 (0.01)	0.512 (0.01)	0.751 (0.01)	0.965 (0.04)	0.844 (0.01)

## Supplementary Materials

The following are available online at https://www.mdpi.com/article/10.3390/s21093139/s1, Supplement S1: Further Descriptions on Machine Learning, Data Analyses and Data Capture, Supplement S2: Patient population details.
