# Supplementary material for: Sensor Validation and Diagnostic Potential of Smartwatches in Movement Disorders
Source: Sensors (Basel). 2021 Apr 30;21(9):3139. doi: 10.3390/s21093139 (PMC8124167; doi:10.3390/s21093139)
Supplement: Supplementary file 1 [file sensors-21-03139-s001.zip › ML-supplement.pdf]

Authors

Julian Varghese, Catharina Marie van Alen, Michael Fujarski, Georg Stefan Schlake, Julitta Sucker, Tobias Warnecke, Christine Thomas

The article 'Sensor Validation and Diagnostic Potential of Smartwatches in Movement Disorders' is a collaborative work of the Geophysics department of the University of Münster, the Institute of Medical Informatics and the neurology department of the University Hostpital Münster. Additionally we want to acknowledge **Julitta Sucker** and **Georg Stefan Schlake** for their contribution on the Deep Learning approaches, especially with the Activity Recognition task.

Supplement

Feature	Description	Complexity
Medical History	Age, height, weight, effect of alcohol on tremor and appearance of PD in kinship. Further details provided on Varghese et al., 2019 (PMID 30761078, supplementary material). Medication and Diagnosis are not used as features as they are too closely linked to the target classes.	-
Symptoms Questionnaire	The number of items that were answered with 'yes' in the Parkinson's Disease Non-Motor Scale by the Movement Disorder Society.	-
Amplitude Distribution 1	Create an Amplitude-Histogram and pick the 30th to 70th percentile in 5 percent steps.	$\mathcal{O}(n \cdot k \cdot log(k))$
Side Dominance 2	Use the 90th percentile from the Amplitude Distribution for the left and right arm. Calculate the ratio. The Ratio is a real number between (0, 1]	$\mathcal{O}(n \cdot k \cdot log(k))$
Standard Deviation of Acceleration	Calculate the Standard Deviation of the raw acceleration data. Each axis has a separate value.	$\mathcal{O}(n \cdot k)$
Fast Fourier Transformation 3	Calculate the 3-dimensional FFT for the assessment step and use a polynomial regression to reduce the output dimensionality. Polynomials of degree 3 are used.	$\mathcal{O}(n \cdot k \cdot log(k))$

Table 1: Used Features with their description. The complexity uses n as the number of samples and k as the number of values in a single task. k equals either 1024 or 2048 datapoints.

Algorithm 1 Amplitude Distribution. The Algorithm shows the procedure for a single time series recording in 3 axes. The actual routine repeats this procedure for each assessment step, for each arm.

```
norm_data ← euclidean_norm([x,y,z] Acceleration)
norm_data ← sort(norm_data)
features ← empty List
for i in 0..8:
    features.append(norm_data[0.3 + i · 0.05])
```

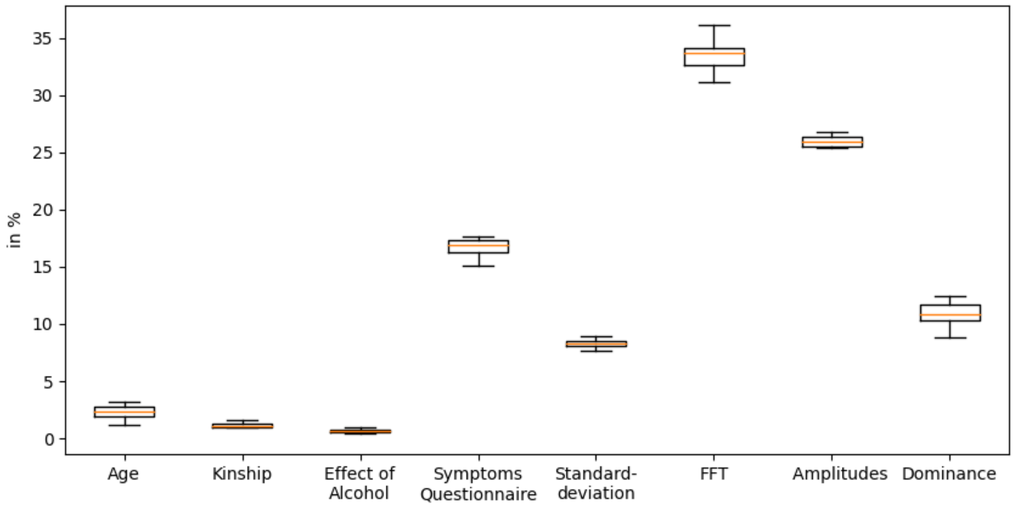

Figure 1: Importances of the Features from Table 1. Importance in percent and calculated by CatBoost.

Algorithm 2 Side Dominance. The Algorithm shows the procedure for a single time series recording in 3 axes. The ratio is calculated with the min/max functions in order to normalize the results to range (0, 1]

norm_data_left ← euclidean_norm([x,y,z] Acceleration-Left)
norm_data_left ← sort(norm_data_left)
norm_data_right ← euclidean_norm([x,y,z] Acceleration-Right)
norm_data_right ← sort(norm_data_right)
dominance ← $\frac{\text{min(norm\_data\_left[0.9], norm\_data\_right[0.9])}}{\text{max(norm\_data\_left[0.9], norm\_data\_right[0.9])}}$

Algorithm 3 Fast Fourier Transformation. The Algorithm shows the procedure for a single timeseries recording in 3 axes. As the FFT produces for our data hundreds of values for each sample we need to reduce the dimensionality. Polynomial regression worked best in this case.

fft_data ← fft([x,y,z] Acceleration)
regression ← poly_regression(fft_data, degree=3)
features ← coefficients(regression)

**Estimator**	**Accuracy**	**Balanced Accuracy**	**Precision**	**Recall**	**F1**
MLP	0.864 (0.03)	0.815 (0.05)	0.907 (0.03)	0.913 (0.03)	0.909 (0.02)
SVM - rbf	0.870 (0.02)	0.827 (0.01)	0.913 (0.01)	0.913 (0.03)	0.913 (0.01)
CatBoost	0.887 (0.02)	0.819 (0.04)	0.901 (0.03)	0.956 (0.03)	0.927 (0.01)
Deep Learning 6	0.768 (0.06)	0.591 (0.07)	0.782 (0.03)	0.954 (0.06)	0.859 (0.04)

Table 2: Average Performances with standard deviations after 5-Fold Crossvalidation for classification of **Parkinson's Disease against healthy subjects**. Standard Deviation (**SD**). Radial Basis Function (**rbf**).

**Estimator**	**Accuracy**	**Balanced Accuracy**	**Precision**	**Recall**	**F1**
MLP	0.823 (0.01)	0.741 (0.03)	0.865 (0.01)	0.905 (0.00)	0.885 (0.00)
SVM - rbf	0.800 (0.02)	0.682 (0.04)	0.831 (0.02)	0.921 (0.01)	0.873 (0.01)
CatBoost	0.817 (0.02)	0.678 (0.03)	0.826 (0.01)	0.956 (0.03)	0.887 (0.01)
Deep Learning 6	0.735 (0.01)	0.512 (0.01)	0.751 (0.01)	0.965 (0.04)	0.844 (0.01)

Table 3: Average Performances with standard deviations after 5-Fold Crossvalidation for classification of **Parkinson's Disease against related movement disorders**. Standard Deviation (**SD**). Radial Basis Function (**rbf**).

**Estimator**	**Accuracy**	**Balanced Accuracy**	**Precision**	**Recall**	**F1**
MLP	0.856 (0.04)	0.772 (0.05)	0.907 (0.02)	0.914 (0.03)	0.910 (0.02)
SVM - rbf	0.838 (0.02)	0.750 (0.03)	0.901 (0.02)	0.897 (0.06)	0.897 (0.02)
CatBoost	0.882 (0.03)	0.757 (0.06)	0.895 (0.02)	0.968 (0.03)	0.929 (0.01)
Deep Learning 6	0.791 (0.03)	0.551 (0.06)	0.814 (0.01)	0.956 (0.03)	0.879 (0.02)

Table 4: Average Performances with standard deviations after 5-Fold Crossvalidation for classification of **all movement disorders against healthy subjects**. Standard Deviation (**SD**). Radial Basis Function (**rbf**).

**Architecture**	**Accuracy (SD)**
**Simple Dense NN**	0.736 (0.013)
**Fully Connected Network**	0.474 (0.063)
**reduced FCN**	0.554 (0.038)
**Residual Network**	**0.786 (0.016)**

Table 5: Classification of 10 assessment steps using deep learning architectures. Performances are the average of a 5-Fold Crossvalidation with their Standard Deviation in brackets.

**Architecture**	**Accuracy**	**Precision**	**Recall**	**F1-Weighted**	**F1**
**Simple Dense NN**	**0.946** (0.011)	0.961 (0.020)	0.930 (0.017)	0.946 (0.011)	0.945 (0.010)
**reduced FCN**	0.896 (0.061)	0.879 (0.083)	0.944 (0.029)	0.892 (0.069)	0.906 (0.044)
**ResNet**	0.935 (0.016)	0.946 (0.027)	0.925 (0.035)	0.935 (0.016)	0.934 (0.016)

Table 6: Classification of the assessment steps DrinkGlass and PointFinger using deep learning architectures. Performances are the average over a 5-Fold Crossvalidation with their standard deviation. FCN = Fully Connected Network.

**Architecture**	**Accuracy (SD)**
**Simple Dense NN**	0.822 (0.012)
**Fully Connected Network**	0.748 (0.130)
**reduced FCN**	0.783 (0.012)
**Residual Network**	**0.837** (0.022)

Table 7: Classification of 5 assessment steps using deep learning architectures. Performances are the average over a 5-Fold Crossvalidation with their standard deviation.

**Estimator**	**Hyperparameter**	**Values**
MLP	hidden_layer_sizes	(70)
		(50)
		(30, 10)
SVM	C	LogRange(-5, 5)
	gamma	LogRange(-5, 5)
CatBoost	Iterations	1000
	max_depth	6
Deep Learning	Epochs	100
	Callbacks	EarlyStopping
	Architectures	(See Figures 1-5)

Table 8: Hyperparameters used for Machine Learning.

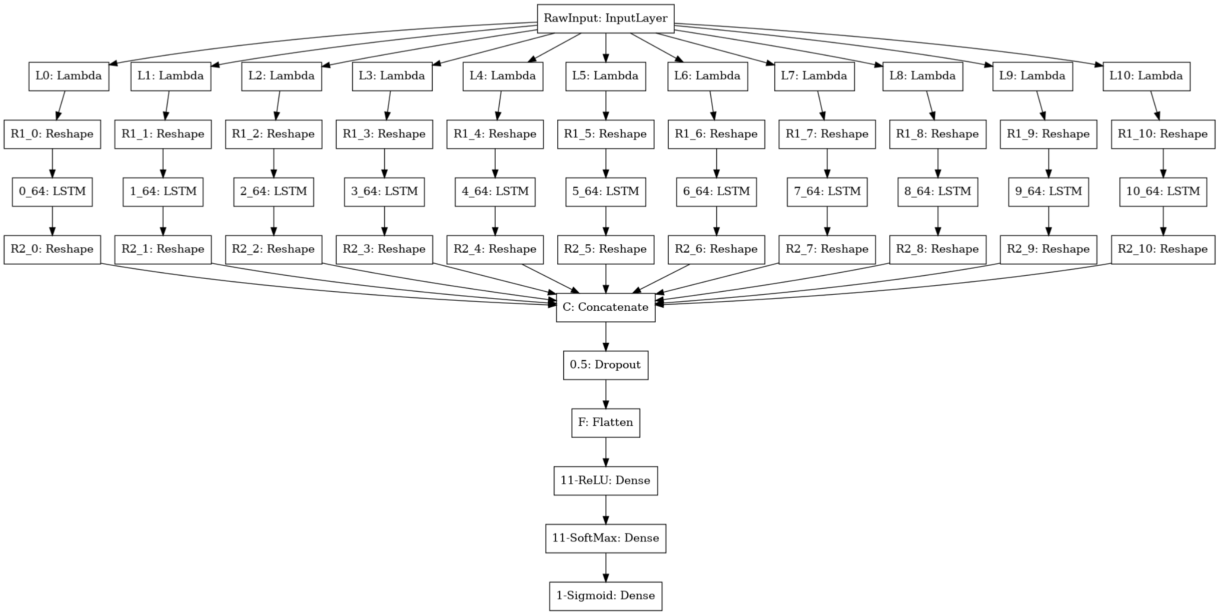

Figure 2: Deep Architecture utilizing LSTM Cells to learn from raw acceleration and rotation input. Lambda-Layers are used to split the several assessment steps of each subject into separate LSTM Branches. The LSTM cells produce 64 outputs each. Both Dense layers produce 11 outputs. The output Dense layer produces a single output between [0, 1]. For an increased readability the shapes of this model are not shown. The input-shape is (11, 1024, 3). Each branch reshapes the data to (64, 16) to process 16 values at once. The output is reshaped to (-1, 1) to process the data with a convolution layer.

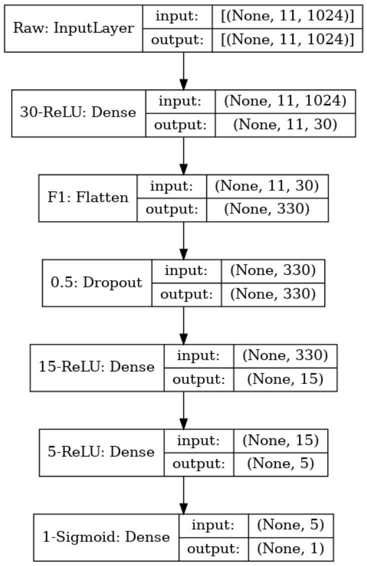

Figure 3: Deep learning architecture with simple dense layers. The shape (11, 1024) results from the 11 assessment steps that were performed by each subject with 1024 datapoints each. This architecture was used with the norm (no channels) and with the x-, y-, z-axis as channels recorded by the sensors, changing the shape to (11, 1024, 3). A shape of (None, ..) describes the unknown number of samples that will be fed to the model.

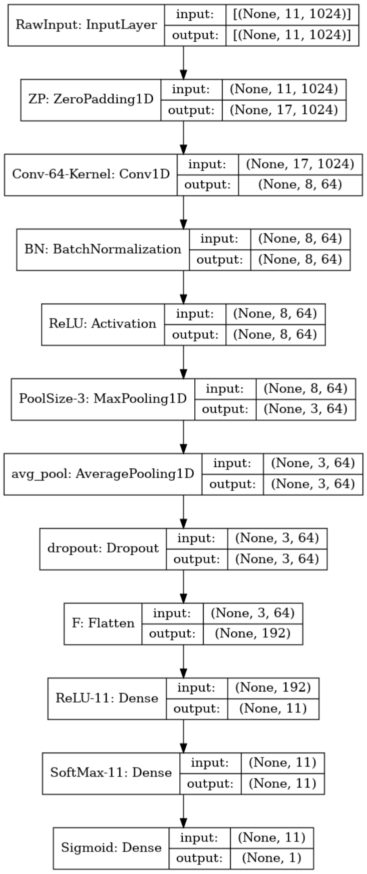

Figure 4: Deep learning architecture with a mixture of convolutions and dense layers. This architecture was used with the norm (no channels) and with the x-, y-, z-axis as channels recorded by the sensors, changing the shape to (11, 1024, 3). A shape of (None, ..) describes the unknown number of samples that will be fed to the model.

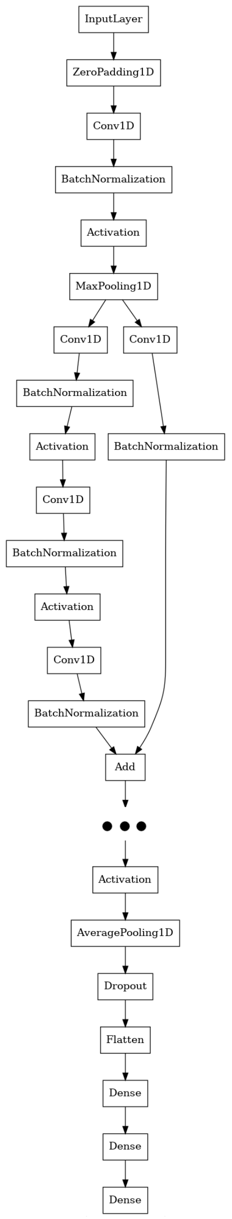

Figure 5: Deep learning architecture with a complex Residual Network. This architecture was used with one to five residual blocks. Shapes of the layers are not shown due to readability. This architecture uses the same shapes as the simpler CNN in Figure 4.

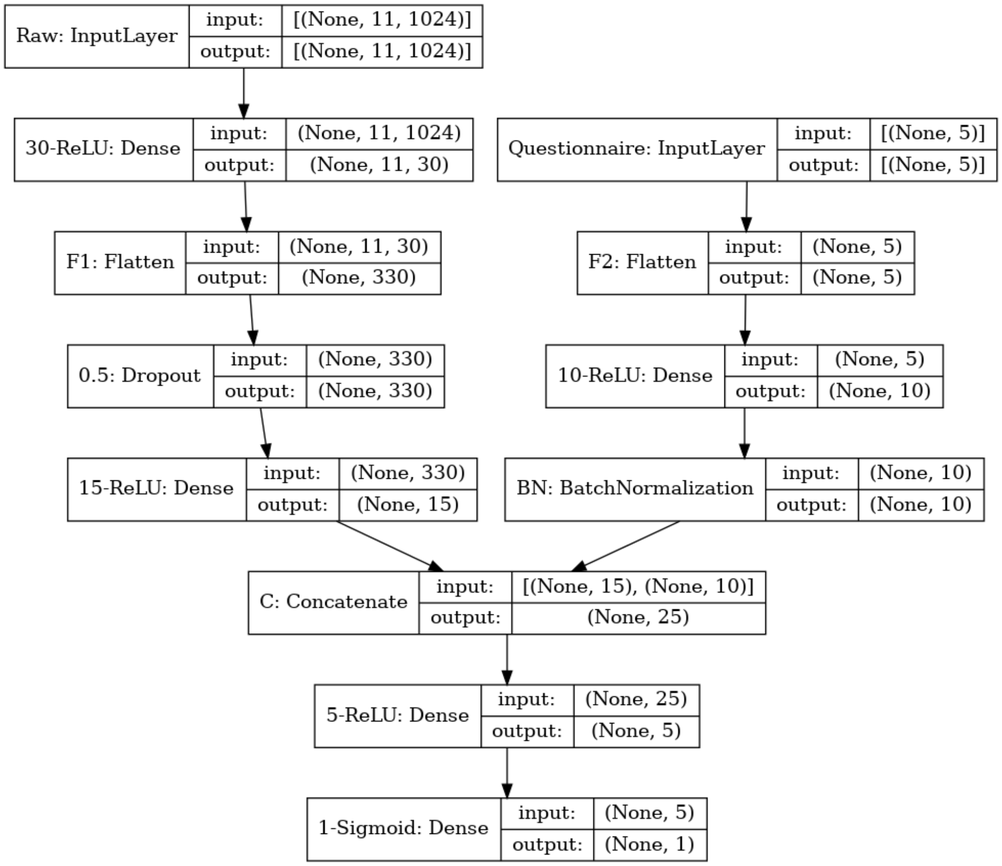

Figure 6: Best performing Deep learning architecture with simple Dense layers. This architecture includes a separate input branch for questionnaire features.

Apple Watch Series	Measurement Day	Sample Rate (Hz)
3	1	99.61
3	2	99.66
4	2	99.40

Table 9: Actual Sample Rates in contrast to the programmed 100Hz.

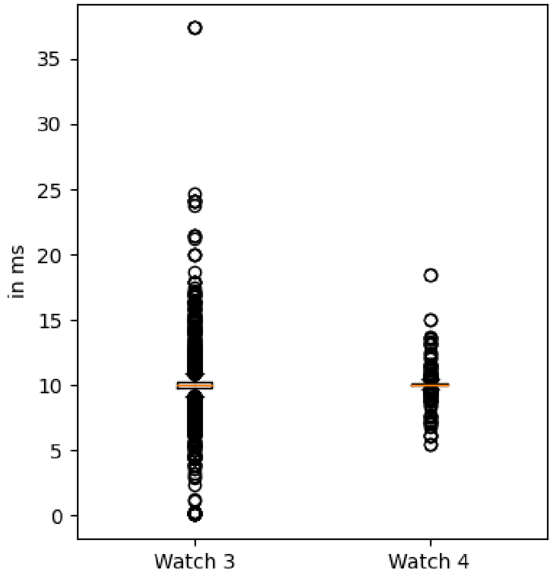

Figure 7: Time in ms between two measured datapoints. Expected value should be at 10ms as the sampling rate was set to 100Hz. Apple Watch Series 4 has a lower standard deviation. Both watches have a mean of roughly 9.95ms as shown in Table 9.
