# Supplementary material for: Sensor Validation and Diagnostic Potential of Smartwatches in Movement Disorders
Source: Sensors (Basel). 2021 Apr 30;21(9):3139. doi: 10.3390/s21093139 (PMC8124167; doi:10.3390/s21093139)
Supplement: Supplementary file 1 [file sensors-21-03139-s001.zip › population.pdf]

Table 1: Common abbreviations for the following population of the clinical trial.

| Abbreviation | Description | Translation |
|---|---|---|
| IPS | Idiopatisches Parkinson Syndrom | Idiopathic Parkinsonism |
| THS | Tiefe Hirnstimulation | Deep Brain Stimulation |
| Z.n. | Zustand nach | Condition after |

| Condition | Disease Comment | Age | Gender |
|---|---|---|---|
| Healthy | | 65 | f |
| Healthy | | 35 | f |
| Healthy | | 49 | m |
| Healthy | | 50 | f |
| Healthy | | 42 | f |
| Healthy | Essential Tremor | 69 | m |
| Healthy | | 56 | m |
| Healthy | Osteoarthritis | 75 | f |
| Healthy | | 54 | f |
| Healthy | | 79 | f |
| Healthy | | 90 | m |
| Healthy | | 88 | f |
| Healthy | | 49 | m |
| Healthy | | 48 | f |
| Healthy | | 80 | m |
| Healthy | | 84 | f |
| Healthy | | 80 | m |
| Healthy | | 67 | f |
| Healthy | Z. n. Breast cancer, Z. n. port associated thrombus, Z.n. Lubar Vertebrae fracture, Hypothyroidism | 52 | f |
| Healthy | | 48 | m |
| Healthy | | 72 | f |
| Healthy | | 54 | f |
| Healthy | | 67 | f |
| Healthy | | 44 | f |
| Healthy | | 56 | m |
| Healthy | | 63 | m |
| Healthy | | 40 | m |
| Healthy | | 56 | m |
| Healthy | | 42 | f |
| Healthy | | 48 | f |
| Healthy | | 58 | f |
| Healthy | | 76 | f |
| Healthy | | 81 | m |
| Healthy | | 76 | f |
| Healthy | | 81 | m |
| Healthy | | 51 | f |
| Healthy | | 61 | f |
| Healthy | | 38 | m |
| Healthy | | 60 | m |
| Healthy | | 64 | m |
| Healthy | Z. n. bacterial Meningitis | 43 | f |
| Healthy | IPS mixed type | 64 | m |
| Healthy | Gait disorder of unknown etiology | 52 | f |
| Healthy | Stiff Limb plus Syndrome | 69 | f |

| | | | |
|---|---|---|---|
| Healthy | Segmental Dystonia and Myoclonus syndrome | 66 | f |
| Healthy | Hypertonia, PCO, Diabetes mellitus II, Asthma | 61 | f |
| Healthy | | 40 | f |
| Healthy | | 67 | f |
| Healthy | deafness right | 70 | m |
| Healthy | | 71 | f |
| Healthy | | 52 | f |
| Healthy | | 45 | m |
| Healthy | | 35 | m |
| Healthy | | 65 | m |
| Healthy | | 85 | m |
| Healthy | | 40 | f |
| Healthy | | 78 | f |
| Healthy | | 61 | f |
| Healthy | | 56 | m |
| Healthy | | 68 | m |
| Healthy | | 72 | f |
| Healthy | | 38 | f |
| Healthy | | 67 | m |
| Healthy | | 55 | f |
| Healthy | | 79 | f |
| Healthy | | 61 | f |
| Healthy | | 69 | f |
| Healthy | | 77 | m |
| Healthy | | 62 | f |
| Healthy | | 61 | f |
| Healthy | | 64 | m |
| Healthy | | 66 | f |
| Healthy | | 58 | f |
| Healthy | Thyroid carcinoma | 54 | f |
| Healthy | Hypertonia | 71 | f |
| Healthy | | 57 | m |
| Healthy | | 49 | f |
| Healthy | | 66 | f |
| Healthy | | 63 | f |
| Healthy | | 70 | f |
| Healthy | | 60 | f |
| Healthy | | 54 | f |
| Healthy | | 83 | m |
| Healthy | | 76 | f |
| Healthy | | 57 | f |
| Healthy | | 45 | f |
| Healthy | | 48 | f |
| Healthy | | 65 | m |
| Parkinson’s | THS IPS tremordominant type | 51 | m |
| Parkinson’s | IPS hypokinetic-rigid type | 70 | m |
| Parkinson’s | IPS mixed type | 68 | m |
| Parkinson’s | IPS akinetic-rigid type | 72 | m |
| Parkinson’s | IPS mixed type | 57 | m |
| Parkinson’s | IPS mixed type. THS | 53 | m |
| Parkinson’s | IPS hypokinetic-rigid type | 78 | m |
| Parkinson’s | IPS hypokinetic-rigid type | 73 | m |
| Parkinson’s | IPS akinetic-rigid type | 72 | m |

| | | | |
|---|---|---|---|
| Parkinson's | IPS mixed type | 78 | m |
| Parkinson's | IPS tremordominant type. V.a. Restless-Legs-Syndrom. THS | 59 | m |
| Parkinson's | IPS hypokinetic-rigid type | 84 | m |
| Parkinson's | IPS akinetic-rigid type | 80 | m |
| Parkinson's | IPS hypokinetic-rigid type | 59 | m |
| Parkinson's | IPS akinetic-rigid type | 51 | m |
| Parkinson's | IPS tremordominant type | 75 | m |
| Parkinson's | | 75 | m |
| Parkinson's | IPS hypokinetic-rigid type | 73 | m |
| Parkinson's | | 75 | m |
| Parkinson's | IPS mixed type. THS | 47 | m |
| Parkinson's | | 55 | f |
| Parkinson's | | 65 | m |
| Parkinson's | Periodic movement of legs while asleep, mostly uncorrelated Arousals. Lewy-Body-Dementia | 59 | m |
| Parkinson's | IPS akinetic-rigid type | 75 | m |
| Parkinson's | IPS akinetic-rigid type | 71 | m |
| Parkinson's | IPS mixed type | 68 | m |
| Parkinson's | IPS akinetic-rigid type | 67 | m |
| Parkinson's | IPS tremordominant type | 59 | m |
| Parkinson's | IPS hypokinetic-rigid type | 53 | f |
| Parkinson's | IPS hypokinetic-rigid type | 56 | f |
| Parkinson's | IPS hypokinetic-rigid type | 74 | m |
| Parkinson's | IPS mixed type. THS. Essential Tremor | 47 | m |
| Parkinson's | IPS akinetic-rigid type | 63 | f |
| Parkinson's | IPS mixed type | 67 | m |
| Parkinson's | IPS hypokinetic-rigid type | 74 | m |
| Parkinson's | IPS hypokinetic-rigid type | 55 | m |
| Parkinson's | IPS akinetic-rigid type | 65 | f |
| Parkinson's | tremordominant type | 75 | f |
| Parkinson's | tremordominant type SWEDD | 56 | m |
| Parkinson's | mixed type. THS | 56 | m |
| Parkinson's | mixed type | 77 | m |
| Parkinson's | IPS hypokinetic-rigid type | 77 | m |
| Parkinson's | IPS | 61 | m |
| Parkinson's | IPS hypokinetic-rigid type | 71 | m |
| Parkinson's | IPS mixed type | 53 | m |
| Parkinson's | IPS tremordominant type. Resting tremor right | 82 | f |
| Parkinson's | IPS hypokinetic-rigid type | 69 | m |
| Parkinson's | iPS hypokinetic-rigid type | 64 | m |
| Parkinson's | IPS hypokinetic-rigid type | 63 | f |
| Parkinson's | IPS hypokinetic-rigid type | 65 | f |
| Parkinson's | IPS hypokinetic-rigid type THS | 73 | f |
| Parkinson's | IPS mixed type | 57 | f |
| Parkinson's | iPS von hypokinetisch rigiden Typ | 71 | f |
| Parkinson's | IPS mixed type | 62 | m |
| Parkinson's | IPS mixed type | 83 | m |
| Parkinson's | IPS hypokinetic-rigid type | 77 | m |
| Parkinson's | IPS hypokinetic-rigid type | 65 | m |
| Parkinson's | IPS mixed type | 64 | m |
| Parkinson's | IPS mixed type | 57 | f |
| Parkinson's | IPS tremordominant type | 58 | f |

| | | | |
|---|---|---|---|
| Parkinson's | IPS hypokinetic-rigid type | 75 | m |
| Parkinson's | IPS mixed type | 74 | m |
| Parkinson's | Multiple System Atrophy of Parkinson-Type | 71 | m |
| Parkinson's | IPS tremordominant type | 71 | m |
| Parkinson's | IPS tremordominant type | 63 | m |
| Parkinson's | IPS mixed type. THS | 61 | f |
| Parkinson's | IPS tremordominant type. Essential Tremor | 78 | m |
| Parkinson's | IPS mixed type. Essential Tremor | 74 | m |
| Parkinson's | IPS hypokinetic-rigid type Hypertonia | 70 | m |
| Parkinson's | IPS akinetic-rigid type | 75 | m |
| Parkinson's | IPS mixed type. Restless-Legs-Syndrom | 83 | f |
| Parkinson's | IPS mixed type | 63 | f |
| Parkinson's | IPS akinetic-rigid type | 67 | f |
| Parkinson's | IPS tremordominant type. Restless Legs-Syndrom | 63 | f |
| Parkinson's | IPS akinetic-rigid type | 79 | f |
| Parkinson's | iPS mixed type | 70 | f |
| Parkinson's | IPS akinetic-rigid type | 54 | f |
| Parkinson's | IPS akinetic-rigid type. THS | 54 | m |
| Parkinson's | IPS tremordominant type DD monosymptomatic tremor | 72 | m |
| Parkinson's | IPS hypokinetic-rigid type | 65 | m |
| Parkinson's | IPS akinetic-rigid type | 54 | m |
| Parkinson's | IPS hypokinetic-rigid type | 74 | m |
| Parkinson's | IPS akinetic-rigid type | 51 | f |
| Parkinson's | IPS mixed type. Restless-Legs-Syndrome | 81 | f |
| Parkinson's | IPS tremordominant type | 69 | m |
| Parkinson's | IPS akinetic-rigid type. THS | 65 | m |
| Parkinson's | IPS mixed type of unknown classification, DD: vascular, idiopathic | 56 | f |
| Parkinson's | IPS tremordominant type | 80 | f |
| Parkinson's | IPS akinetic-rigid type | 66 | f |
| Parkinson's | IPS mixed type. Essential Tremor | 88 | m |
| Parkinson's | IPS, not yet medicated | 55 | f |
| Parkinson's | IPS tremordominant type | 67 | m |
| Parkinson's | | 49 | m |
| Parkinson's | IPS tremordominant type. Restless-Legs-Syndrome | 58 | m |
| Parkinson's | IPS akinetc rigid type | 55 | f |
| Parkinson's | IPS mixed type | 65 | m |
| Parkinson's | IPS vom akinetc rigid type | 74 | m |
| Parkinson's | IPS mixed type | 68 | m |
| Parkinson's | IPS vom hypokinetc rigid type | 78 | f |
| Parkinson's | IPS mixed type | 79 | f |
| Parkinson's | IPS mixed type | 65 | f |
| Parkinson's | IPS mixed type THS | 49 | m |
| Parkinson's | IPS mixed type. THS | 69 | m |
| Parkinson's | IPS with dystonia | 42 | m |
| Parkinson's | IPS akinetc rigid type | 78 | m |
| Parkinson's | IPS mixed type | 68 | f |
| Parkinson's | IPS tremordominant type | 61 | m |
| Parkinson's | IPS tremordominant type | 73 | m |
| Parkinson's | IPS mixed type | 70 | f |
| Parkinson's | IPS mixed type | 68 | m |
| Parkinson's | Vascular L-Dopa-responsives. hypokinetic rigid parkinsonism | 67 | m |
| Parkinson's | IPS tremordominant type | 72 | m |

| | | | |
|---|---|---|---|
| Parkinson's | IPS hypokinetic-rigid type | 77 | m |
| Parkinson's | IPS tremordominant type | 68 | m |
| Parkinson's | IPS hypokinetic-rigid type | 64 | m |
| Parkinson's | IPS mixed type | 69 | f |
| Parkinson's | IPS hypokinetic-rigid type, z. N. endocarditis | 66 | m |
| Parkinson's | IPS vom akinetisch rigider Typ | 64 | m |
| Parkinson's | IPS tremordominant type | 60 | m |
| Parkinson's | IPS mixed type | 79 | m |
| Parkinson's | IPS vom akinetisch rigider Typ | 69 | m |
| Parkinson's | IPS akinetic-rigid type | 68 | m |
| Parkinson's | Ips tremordominant type | 81 | m |
| Parkinson's | Ips hypokinetic-rigid type THS | 56 | m |
| Parkinson's | IPS akinetic-rigid type | 78 | f |
| Parkinson's | Starting IPS tremordominant type | 47 | m |
| Parkinson's | | 70 | m |
| Parkinson's | Ips mixed type | 73 | f |
| Parkinson's | Ips mixed type THS | 78 | m |
| Parkinson's | IPS hypokinetic-rigid type | 51 | f |
| Parkinson's | IPS tremordominant type | 78 | m |
| Parkinson's | Atypical Parkinsonism with orthostatic dysregulation | 74 | m |
| Parkinson's | IPS tremordominant type | 62 | m |
| Parkinson's | IPS akinetic-rigid type | 63 | m |
| Parkinson's | IPS hypokinetic-rigid type | 73 | m |
| Parkinson's | IPS hypokinetic-rigid type | 74 | m |
| Parkinson's | IPS akinetic-rigid type | 78 | m |
| Parkinson's | IPS mixed type. Severely pronounced Restless-Legs-Syndrome. THS | 68 | m |
| Parkinson's | IPS mixed type | 66 | m |
| Parkinson's | IPS mixed type | 64 | m |
| Parkinson's | Hypertonie | 87 | m |
| Parkinson's | IPS hypokinetic-rigid type | 47 | m |
| Parkinson's | IPS hypokinetic-rigid type | 75 | m |
| Parkinson's | IPS mixed type. Restless-Legs-Syndrome | 67 | f |
| Parkinson's | IPS tremordominant type | 66 | m |
| Parkinson's | IPS mixed type | 67 | m |
| Parkinson's | Parkinsonism with right-sided rigor and tremor of the upper extremities | 76 | m |
| Parkinson's | IPS mixed type | 66 | m |
| Parkinson's | IPS akinetic-rigid type | 49 | m |
| Parkinson's | IPS akinetic-rigid type | 67 | m |
| Parkinson's | IPS mixed type Ausgeprägte orthostatische Dysregulation | 73 | f |
| Parkinson's | Hypokinetic-rigid Type. Left-sided. DD atypical parkinsonism | 63 | f |
| Parkinson's | IPS mixed type | 75 | m |
| Parkinson's | IPS mixed type | 53 | m |
| Parkinson's | IPS mixed type | 55 | m |
| Parkinson's | IPS mixed type | 62 | m |
| Parkinson's | IPS tremordominant type. Symptomatic Restless-Legs-Syndrome | 85 | m |
| Parkinson's | IPS mixed type | 74 | f |
| Parkinson's | Ips tremordominant type | 71 | m |
| Parkinson's | IPS tremordominant type | 66 | f |

| | | | |
|---|---|---|---|
| Parkinson's | IPS hypokinetic-rigid type, no tremor. THS | 58 | f |
| Parkinson's | IPS tremordominant type, distinctive tremor in whole left arm. THS. The device for THS was deactivated before measurement | 46 | m |
| Parkinson's | IPS akinetic-rigid type. THS | 54 | f |
| Parkinson's | IPS mixed type | 69 | m |
| Parkinson's | IPS mixed type. Paralysis of the right arm | 62 | m |
| Parkinson's | IPS mixed type THS | 63 | m |
| Parkinson's | IPS mixed type | 71 | m |
| Parkinson's | IPS akinetic-rigid type | 71 | m |
| Parkinson's | Ips akinetic-rigid type. THS | 75 | f |
| Parkinson's | IPS mixed type | 78 | m |
| Parkinson's | IPS mixed type | 78 | m |
| Parkinson's | IPS hypokinetic-rigid type | 55 | m |
| Parkinson's | IPS tremordominant type | 50 | f |
| Parkinson's | IPS tremordominant type | 54 | f |
| Parkinson's | IPS hypokinetic-rigid type | 70 | m |
| Parkinson's | IPS akinetic-rigid type | 56 | m |
| Parkinson's | IPS mixed type. THS | 58 | m |
| Parkinson's | iPS hypokinetic-rigid type | 60 | f |
| Parkinson's | Ips akinetic-rigid type | 59 | m |
| Parkinson's | Ips akinetic-rigid type | 66 | m |
| Parkinson's | IPS hypokinetic-rigid type | 67 | m |
| Parkinson's | IPS tremordominant type. Severe secondary Restless-Legs-Syndrome | 65 | m |
| Parkinson's | IPS mixed type. THS | 78 | m |
| Parkinson's | IPS hypokinetic-rigid type | 47 | f |
| Parkinson's | IPS hypokinetic-rigid type | 69 | m |
| Parkinson's | IPS mixed type | 80 | m |
| Parkinson's | IPS akinetic-rigid type | 78 | m |
| Parkinson's | IPS akinetic-rigid type | 64 | f |
| Parkinson's | IPS akinetic-rigid type | 70 | m |
| Parkinson's | IPS hypokinetic-rigid type. THS | 51 | m |
| Parkinson's | IPS hypokinetic-rigid type | 70 | m |
| Parkinson's | IPS mixed type. Periodic movement of legs while asleep | 74 | m |
| Parkinson's | IPS tremordominant type | 53 | m |
| Parkinson's | IPS hypokinetic-rigid type | 53 | m |
| Parkinson's | IPS akinetic-rigid type | 46 | f |
| Parkinson's | IPS. Secondary progressive multiple sklerosis | 57 | m |
| Parkinson's | IPS mixed type | 73 | m |
| Parkinson's | IPS hypokinetic-rigid type. THS | 67 | m |
| Parkinson's | IPS mixed type | 73 | m |
| Parkinson's | IPS mixed type | 66 | m |
| Parkinson's | IPS mixed type. THS | 66 | m |
| Parkinson's | IPS tremordominant type | 64 | m |
| Parkinson's | IPS hypokinetic-rigid type. THS | 61 | m |
| Parkinson's | IPS mixed type | 52 | m |
| Parkinson's | IPS tremordominant type | 54 | f |
| Parkinson's | IPS mixed type | 61 | m |
| Parkinson's | IPS tremordominant type | 72 | f |
| Parkinson's | IPS tremordominant type. THS | 51 | m |
| Parkinson's | IPS mixed type | 65 | m |
| Parkinson's | IPS hypokinetic-rigid type | 56 | m |
| Parkinson's | IPS tremordominant type | 72 | m |

| | | | |
|---|---|---|---|
| Parkinson's | IPS tremordominant type | 53 | m |
| Parkinson's | IPS tremordominant type | 67 | f |
| Parkinson's | IPS mit zunehmender Gangstörung und Sturzneigung | 65 | m |
| Parkinson's | IPS tremordominant type | 75 | m |
| Parkinson's | IPS tremordominant type | 77 | f |
| Parkinson's | IPS hypokinetic-rigid type. THS | 61 | m |
| Parkinson's | IPS hypokinetisch rigiden Typ | 69 | m |
| Parkinson's | Ips hypokinetisch rigiden Typ | 49 | f |
| Parkinson's | Ips mixed type | 79 | f |
| Parkinson's | IPS tremordominant type | 77 | m |
| Parkinson's | IPS akinetic-rigid type | 69 | f |
| Parkinson's | IPS mixed type | 72 | m |
| Parkinson's | IPS tremordominant type | 70 | f |
| Parkinson's | IPS tremordominant type | 75 | m |
| Parkinson's | IPS hypokinetisch rigiden Typ | 81 | m |
| Parkinson's | IPS DD MSA | 63 | m |
| Parkinson's | IPS akinetic rigid type | 58 | f |
| Parkinson's | IPS mixed type | 70 | f |
| Parkinson's | | 63 | m |
| Parkinson's | IPS + THS | 48 | m |
| Parkinson's | IPS Tremordominanztyp mit THS | 55 | f |
| Parkinson's | iPS hypokinetic-rigid type. THS before optimization | 61 | m |
| Parkinson's | IPS hypokinetic-rigid type THS | 49 | f |
| Parkinson's | IPS DD Essential Tremor | 69 | m |
| Parkinson's | IPS tremordominant type | 42 | f |
| Parkinson's | IPS mixed type | 76 | m |
| Parkinson's | IPS akinetic-rigid type, THS, Duodopapump | 57 | f |
| Parkinson's | IPS hypokinetic rigid type | 72 | m |
| Parkinson's | Z.n. Operation after spinal disc herniation Z.n. Hemilaminectomy and revision Z.n. Fracture of humerus head right | 66 | m |
| Parkinson's | Diabetes mellitus. Polyneuropathy. Restless-Legs syndrome | 73 | f |
| Parkinson's | IPS mixed type,THS: ja | 50 | m |
| Parkinson's | IPS hypokinetic-rigid type. Test of Duodopapump | 76 | f |
| Parkinson's | IPS tremordominant type. Z.n. THS | 56 | m |
| Parkinson's | IPS mixed type | 71 | m |
| Parkinson's | IPS hypokinetic-rigid type | 69 | m |
| Parkinson's | IPS hypokinetic-rigid type. Z.n. Duodopapump | 72 | f |
| Parkinson's | Starting IPS | 85 | m |
| Parkinson's | IPS hypokinetic-rigid type, full Dyskinesia | 67 | f |
| Parkinson's | | 76 | m |
| Parkinson's | Multiple System Atrophy of Parkinson-Type | 64 | m |
| Parkinson's | Multiple System Atrophy of Parkinson-Type | 61 | f |
| Parkinson's | IPS akinetic-rigid Type | 68 | f |
| Parkinson's | IPS akinetic-rigid type | 66 | m |
| Parkinson's | Essential Tremor DD IPS | 69 | m |
| Parkinson's | Essential Tremor DD IPS | 69 | m |
| Essential Tremor | Essential Tremor | 57 | m |
| Essential Tremor | Essential Tremor. THS | 70 | m |
| Essential Tremor | Essential Tremor | 78 | f |
| Essential Tremor | Essential Tremor. THS | 59 | f |
| Essential Tremor | Essential Tremor | 63 | m |

| | | | |
|---|---|---|---|
| Essential Tremor | Essential Tremor | 69 | m |
| Essential Tremor | Essential Tremor | 54 | m |
| Essential Tremor | ET THS | 79 | m |
| Essential Tremor | ET and oromandibular dystonia. Risperidon-associated | 70 | m |
| Essential Tremor | Essential Tremor of head and hands. THS | 61 | f |
| Essential Tremor | Essential Tremor | 64 | f |
| Essential Tremor | Essential Tremor | 72 | m |
| Essential Tremor | Essential Tremor. Periodic leg movement while awake and asleep. Restless-Legs-Syndrome during iron deficiency | 72 | m |
| Essential Tremor | THS | 61 | m |
| Essential Tremor | | 52 | f |
| Essential Tremor | THS | 82 | m |
| Essential Tremor | | 79 | f |
| Essential Tremor | | 64 | m |
| Essential Tremor | | 45 | f |
| Essential Tremor | | 77 | f |
| Essential Tremor | | 49 | m |
| Essential Tremor | | 62 | m |
| Essential Tremor | Z.n. THS. Before optimization of THS | 50 | f |
| Essential Tremor | Z.n. THS. Before optimization of THS | 78 | f |
| Essential Tremor | Temporal lobe epilepsy | 57 | f |
| Essential Tremor | ET since he remembers. Diagnosed 08/2018. Additionally Alzheimer's Disease | 64 | m |
| Other Movement Disorders | Swedd | 41 | f |
| Other Movement Disorders | multiple sklerosis | 67 | f |
| Other Movement Disorders | MSA-C | 67 | f |
| Other Movement Disorders | Idiopathic generalized dystonia. THS | 66 | f |
| Other Movement Disorders | Tardive dyskinesia after neuroleptic therapy | 53 | f |
| Other Movement Disorders | Dystonia-Parkinson-Syndrome, maybe genetically | 51 | m |
| Other Movement Disorders | Tremor with unknown cause DD Alzheimer's Disease | 83 | f |
| Other Movement Disorders | Atypical PD DD PSP | 79 | m |
| Other Movement Disorders | Multiple System Atrophy of Parkinson-Type (MSA-P) | 58 | m |
| Other Movement Disorders | L-Dopa-responsive dystonia | 51 | f |
| Other Movement Disorders | Left-Sided resting tremor and hypokinesia without presynaptic striatal deficit | 81 | m |
| Other Movement Disorders | Functional movement disorder. Likely caused by chronic pain syndrome | 33 | m |
| Other Movement Disorders | Lewy-Body disease with posture tremor | 73 | m |
| Other Movement Disorders | Starting IPS DD age dependent tremor DD asymmetric Essential Tremor | 69 | m |
| Other Movement Disorders | MSA of cerebellar type | 67 | m |
| Other Movement Disorders | Drug-induced tremor. Maybe Valproat | 60 | f |
| Other Movement Disorders | Dystonial tremor of the head. Anamnestic also generalized tremor/dystonia | 41 | f |
| Other Movement Disorders | Hypoxic brain damage in childhood with symptomatic generalized dystonia, left sided. THS | 52 | m |
| Other Movement Disorders | Idiopathic polyneuropathy with ataxy and tremor - DD hereditary DD inflammatory | 65 | f |
| Other Movement Disorders | Leukoencephalopathy of unknown etiology | 48 | f |
| Other Movement Disorders | Maybe normal-pressure-hydrocephalus. DD starting IPS | 63 | m |
| Other Movement Disorders | Paroxysmal kinesigenic dyskinesia | 33 | f |

| | | | |
|---|---|---|---|
| Other Movement Disorders | IPS akinetic-rigid type DD PSP of Parkinson type | 77 | m |
| Other Movement Disorders | Asymmetric tremor of the hand of unknown etiology | 59 | f |
| Other Movement Disorders | Progressive supranuclear conjugate gaze palsy a.e. type Richardson. Restless Legs Syndrome | 74 | f |
| Other Movement Disorders | Segmental dystonia. THS | 46 | f |
| Other Movement Disorders | Restless-Legs-Syndrome | 69 | f |
| Other Movement Disorders | Multiple Sklerosis | 55 | m |
| Other Movement Disorders | Spinocerebellar syndrome with cerebral atrophy. intention tremor both-sided asymmetric | 69 | m |
| Other Movement Disorders | Dystonic tremor | 85 | f |
| Other Movement Disorders | Segmental dystonia | 60 | m |
| Other Movement Disorders | Multiple System Atrophy of Combined-Type | 77 | f |
| Other Movement Disorders | Cervical dystonia | 68 | m |
| Other Movement Disorders | Multiple Sklerosis of relapsing type | 49 | f |
| Other Movement Disorders | Dystonia of unknown etiology | 51 | f |
| Other Movement Disorders | Left-sided tremor. Maybe increased physiological tremor | 69 | m |
| Other Movement Disorders | Clinically diagnosed L-Dopa responsive dystonia | 52 | f |
| Other Movement Disorders | Multiple Sklerosis of relapsing type | 37 | m |
| Other Movement Disorders | MSA of Parkinson type | 67 | f |
| Other Movement Disorders | Mild cervical dystonia | 55 | f |
| Other Movement Disorders | Generalized dystonic movement disorder. THS | 35 | f |
| Other Movement Disorders | Dystonic movement disorder. Tremor of the head. Torticollis to he right. THS | 56 | f |
| Other Movement Disorders | Chorea minor Sydenham | 48 | f |
| Other Movement Disorders | Multiple Sklerosis | 51 | m |
| Other Movement Disorders | Multiple Sklerosis of relapsing type | 54 | m |
| Other Movement Disorders | Multiple Sklerosis of primary-progressive type | 62 | m |
| Other Movement Disorders | Multiple Sklerosis of relapsing type | 60 | f |
| Other Movement Disorders | Cervical dystonia with rotatory Torticollis to the right side and Retrocollis. THS | 69 | m |
| Other Movement Disorders | Cervical dystonia with Torticollis with tremor. Essential Tremor | 72 | m |
| Other Movement Disorders | Adult multifocal dystonia of unknown etiology | 44 | f |
| Other Movement Disorders | Cervical dystonia. THS | 56 | f |
| Other Movement Disorders | Multiple Sklerosis of secondary progressive type | 40 | m |
| Other Movement Disorders | Segmental orofacial dystonia with blepharospasm. THS | 77 | f |
| Other Movement Disorders | Torticollis to the left with dystonic tremor | 54 | f |
| Other Movement Disorders | | 45 | f |
| Other Movement Disorders | | 53 | m |
| Other Movement Disorders | Torticolis to the left with dystonic tremor | 54 | f |
| Other Movement Disorders | Unknown Tremor, especially of the head | 80 | f |
| Other Movement Disorders | Coarse resting tremor right with unknown cause | 56 | f |
| Other Movement Disorders | ET DD functional tremor right arm and mild tremor of the head | 42 | f |
| Other Movement Disorders | Asthma bronchiale Z.n. Pericardial effusion | 21 | f |
| Other Movement Disorders | Right dominant tremor. Maybe Lithium-associated | 68 | m |
| Other Movement Disorders | Cervical dystonia | 69 | m |
